# Interplay between Oxidative Stress and Metabolic Derangements in Non-Alcoholic Fatty Liver Disease: The Role of Selenoprotein P

**DOI:** 10.3390/ijms21228838

**Published:** 2020-11-22

**Authors:** Gian Paolo Caviglia, Chiara Rosso, Angelo Armandi, Melania Gaggini, Fabrizia Carli, Maria Lorena Abate, Antonella Olivero, Davide Giuseppe Ribaldone, Giorgio Maria Saracco, Amalia Gastaldelli, Elisabetta Bugianesi

**Affiliations:** 1 Department of Medical Sciences, University of Turin, 10126 Turin, Italy; gianpaolo.caviglia@unito.it (G.P.C.); angelo.armandi@unito.it (A.A.); marialorena.abate@unito.it (M.L.A.); antonella.olivero@unito.it (A.O.); davrib_1998@yahoo.com (D.G.R.); giorgiomaria.saracco@unito.it (G.M.S.); elisabetta.bugianesi@unito.it (E.B.); 2 Cardiometabolic Risk Unit, Institute of Clinical Physiology, CNR, 56121 Pisa, Italy; mgaggini@ifc.cnr.it (M.G.); fabrycarli@hotmail.it (F.C.); amalia@ifc.cnr.it (A.G.); 3 Division of Gastroenterology, Città della Salute e della Scienza University-Hospital, 10100 Turin, Italy

**Keywords:** selenoprotein P, non-alcoholic fatty liver disease, hepatokines, insulin resistance

## Abstract

**Background:** Pathogenetic mechanisms involved in the progression of non-alcoholic fatty liver disease (NAFLD) are complex and multifactorial. We investigated oxidative stress through the measurement of selenoprotein P (SeP) in serum and we explored its relation to metabolic derangements and liver damage in a group of non-diabetic NAFLD subjects. **Methods:** 57 NAFLD patients underwent a double-tracer oral glucose tolerance test (OGTT). Insulin resistance (IR) components were calculated at baseline as follows: hepatic-IR = (endogenous glucose production*insulin); peripheral-IR = (glucose rate of disappearance(Rd)); adipose-tissue(AT)-IR as Lipo-IR = (glycerol rate of appearance (Ra)*insulin) or AT-IR = (free fatty acids (FFAs)*insulin). The lipid and amino acid (AA) profiles were assessed by gas chromatography–mass spectrometry. SeP levels were measured by enzyme immunosorbent assay. **Results:** Circulating SeP correlated with insulin (r_S_ = 0.28), FFAs (r_S_ = 0.42), glucose Rd (r_S_ = −0.33) and glycerol Ra (r_S_ = −0.34); consistently, SeP levels correlated with Lipo-IR and AT-IR (r_S_ > 0.4). Among the AA and lipid profiles, SeP inversely correlated with serine (r_S_ = −0.31), glycine (r_S_ = −0.44) and branched chain AA (r_S_ = −0.32), and directly correlated with saturated (r_S_ = 0.41) and monounsaturated FFAs (r_S_ = 0.40). Hepatic steatosis and fibrosis increased in subjects with higher levels of SeP. In multivariable regression analysis, SeP was associated with the degree of hepatic fibrosis (t = 2.4, *p* = 0.022). **Conclusions:** SeP levels were associated with an altered metabolic profile and to the degree of hepatic fibrosis, suggesting a role in the pathogenesis of NAFLD.

## 1. Introduction

Non-alcoholic fatty liver disease (NAFLD) has become the most common liver disease in Western countries alongside the increased incidence of obesity, type 2 diabetes mellitus (T2DM) and metabolic syndrome. NAFL is a clinical benign condition in which the accumulation of fat in the hepatocytes exceeds 5%. The progressive form, non-alcoholic steatohepatitis (NASH), is characterized by inflammation and ballooning degeneration, [1,2], ultimately progressing to fibrosis, cirrhosis and potentially hepatocellular carcinoma [3,4].

Pathogenetic mechanisms involved in the onset and progression of NAFLD are complex and multifactorial [5]. It is believed that the excessive accumulation of triglycerides in the hepatocytes sensitizes the liver to multiple “hits”, promoting inflammation and fibrogenesis through oxidative stress [5]. Moreover, insulin resistance (IR) has been considered one of the major players in the worsening of liver disease [6]. Briefly, the impaired insulin action in an insulin-resistant state promotes endogenous glucose production by the liver and limits glucose uptake by peripheral tissues (adipose tissue and muscle) resulting in hyperglycemia and compensatory hyperinsulinemia. In the adipose tissue, the impaired lipolysis, due to adipose tissue IR, determines an increased flux of free fatty acids (FFAs) that reach the liver from the adipocytes and promote steatosis and thus enhances oxidative stress [7,8,9,10]. The association between oxidative stress and hepatic fibrosis has been reported both in vitro and in vivo [11,12].

In addition to the alterations in glucose and lipid metabolism, other metabolic derangements are known to impact the progression of liver damage in the setting of NAFLD. Increased plasma concentrations of certain AA, such as branched chain AA (BCAA), have been implicated in the onset and progression of hepatic fibrosis [13,14]; similarly, alterations in circulating levels of glycine, serine and glutamate are implicated in the response to oxidative stress through the synthesis of glutathione [14].

In recent years, there has been considerable interest in the endocrine function of the liver, which, under stress conditions, is able to release several secretory proteins termed hepatokines [15]. Selenoprotein P (SeP) is a liver-derived selenium carrier protein with antioxidant properties that seem to have a role in the development of IR and T2DM. It is well known that SeP is tightly related to glucose metabolism; specifically, high glucose concentration stimulates insulin secretion by the pancreas and SeP release by the liver that in turn exacerbates insulin secretion and hyperinsulinemia contributing to IR. Several studies have shown increased levels of SeP in obese subjects [15,16,17] as well as in patients with T2DM and NAFLD [15,18,19], but its putative role in the complex pathogenesis of NASH has not yet been fully elucidated. The aim of this study was to investigate oxidative stress through the measurement of SeP in serum and to explore its relation with metabolic derangements and liver damage in a well characterized group of non-diabetic subjects with NAFLD.

## 2. Results

The anthropometric, biochemical, and histological characteristics of the study cohort (*n* = 57, median age 43 years) are reported in Table 1. The whole cohort has been grouped according to SeP tertile and the 75th percentile has been used as the higher cut-off value.

Overall, 28% of the patients had a body mass index (BMI) > 29.9 kg/m^2^; however, central obesity assessed by waist circumference (≥ 102 cm in male, ≥ 88 cm in female) was found in almost a half of the study cohort (42%). As per selection criteria, no patients had overt T2DM or dyslipidemia.

Among histological features, hepatic steatosis was mild (< 33%) in 51% of patients, moderate (33–66%) in 35% and severe (≥ 66%) in 14%. A diagnosis of NASH according to the joint presence of hepatic steatosis, ballooning and lobular inflammation was made in the majority of the patients (74%), even if advanced fibrosis (F ≥ 2) and severe fibrosis (F ≥ 3) were found in 57% and 27% of the cases, respectively (Table 1).

According to the SeP cut-off, we found that subjects with SeP levels higher or equal to 11.8 ng/mL had higher BMI and higher AST levels compared to those with lower SeP levels, Table 1. Moreover, we found a sexual dimorphic expression of SeP, which is significantly higher in females compared to males (13.7 ng/mL *versus* 10.1 ng/mL, *p* < 0.001).

### 2.1. Circulating Selenoprotein P Levels, Metabolic Parameters and Insulin Resistance

The correlation between SeP levels and metabolic parameters is reported in Table 2.

**Figure 1:**
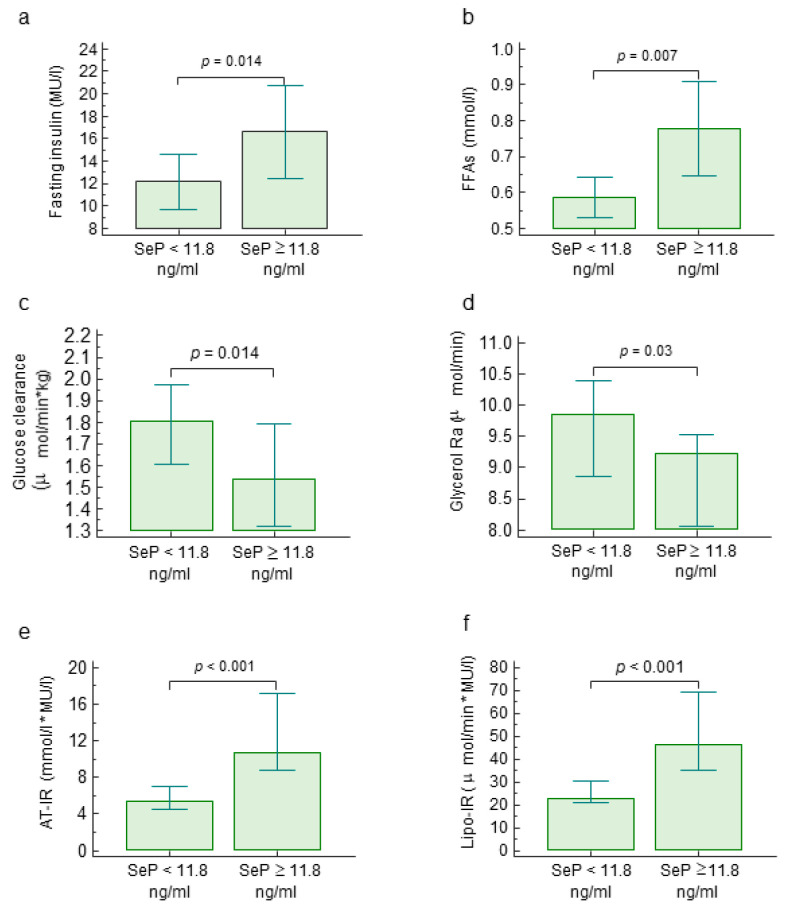
Metabolic parameters and insulin resistance components according to selenoprotein *p* values. Fasting insulin levels (**a**), free fatty acid (FFA) levels (**b**), glucose clearance (**c**) lipolysis (**d**), adipose tissue insulin resistance (IR) by FFAs (**e**) and by glycerol rate of appearance (Ra) (**f**) are reported according to selenoprotein P cut-off, corresponding to the 75th percentile (11.8 ng/mL). Abbreviations: adipose tissue (AT), free fatty acids (FFAs), insulin resistance (IR), rate of appearance (Ra), selenoprotein P (SeP).

Circulating SeP directly correlated with insulin (r_S_ = 0.028, *p* = 0.035) and FFAs levels while inversely correlated with glucose clearance and lipolysis. Accordingly, insulin and FFAs levels were significantly higher in patients with SeP levels higher or equal to 11.8 ng/mL (Figure 1a,b), while glucose clearance and lipolysis significantly decreased in patients with SeP levels higher or equal to 11.8 ng/mL (Figure 1c,d). Among IR components, we found a poor correlation between circulating SeP and homeostasis model of assessment (HOMA)-IR as well as hepatic (Hep)-IR (Table 2) but we observed a strong direct correlation between plasma SeP levels and both adipose tissue insulin resistance (AT-IR) and Lipo-IR (Figure 1e,f), suggesting that SeP is more related to peripheral (muscle and adipose tissue) than hepatic IR (Table 2).

### 2.2. Circulating Selenoprotein P, Amino Acid Profile and Lipid Profile

We further measured plasma concentration of AA to assess their correlation with circulating SeP. We found an inverse correlation between SeP levels and both plasma glycine and serine (r_S_ = −0.44, *p* < 0.001 and r_S_ = −0.31, *p* = 0.017, respectively), two AA involved in glutathione synthesis. Glutathione is one of the major antioxidant systems activated in the neutralization of reactive oxygen species; the decrease in the substrates involved in its synthesis could be related with an increased request of selenium for proper functioning. Similarly, we found an inverse correlation between SeP and BCAA (leucine + isoleucine + valine levels) (r_S_ = −0.32, *p* = 0.015), a group of AA previously correlated to IR, inflammation and fibrosis in patients with chronic hepatitis [9,14,20].

The composition of FFAs was determined in a subgroup of 42 patients. The correlation between SeP levels and FFAs is reported in Table 3.

**Figure 2:**
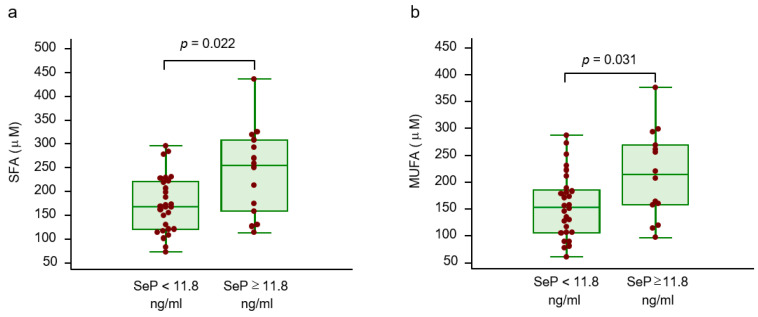
Saturated (**a**) and monounsaturated (**b**) fatty acid levels are reported according to selenoprotein P cut-off, corresponding to the 75th percentile (11.8 ng/mL). Abbreviations: monounsaturated free fatty acids (MUFA), selenoprotein P (SeP), saturated free fatty acids (SFA).

Overall, SeP levels were more correlated to saturated fatty acids (SFAs) and monounsaturated fatty acids (MUFA) compared to polyunsaturated fatty acids (PUFA). Specifically, the stronger correlations were found with palmitic acid, margaric acid and palmitoleic acid (Table 3). Consistently, SFA and MUFA levels were significantly increased in patients with SeP levels higher or equal to 11.8 ng/mL (Figure 2a,b).

### 2.3. Selenoprotein P Levels and Histological Features

**Figure 3:**
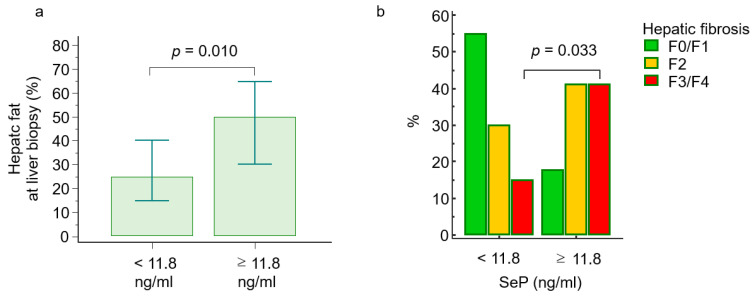
Hepatic steatosis (**a**) and fibrosis (**b**) according to selenoprotein P cut-off. The cut-off value for selenoprotein P corresponds to the 75th percentile (11.8 ng/mL). Abbreviations: fibrosis (F), selenoprotein P (SeP).

Among the histological features of NASH, the amount of liver steatosis, but not the rate of ballooning and lobular inflammation, significantly increased in patients with SeP levels higher or equal to 11.8 ng/mL compared to the counterpart (mean values, 50% *versus* 31%, *p* = 0.010; Figure 3a). Similarly, severe liver fibrosis (F3/F4) was more prevalent in patients with higher levels of SeP (41% *versus* 15%, Figure 3b). Consistently, SeP levels significantly correlated with both hepatic steatosis and the degree of liver fibrosis (r_S_ = 0.26, *p* = 0.048 and r_S_ = 0.41, *p* = 0.002, respectively).

To evaluate the association between SeP levels and the degree of liver fibrosis, we performed a multivariable regression analysis adjusted for age, sex, and BMI. Since several studies in patients with NAFLD have reported a significant association between IR in the adipose tissue and hepatic fibrosis, we included the AT-IR index in the regression model. As reported in Table 4, SeP levels, but not AT-IR, were significantly associated with the degree of liver fibrosis (t = 2.37, *p* = 0.022) suggesting that the increase in SeP levels, consequent to an increased response to the oxidative stress and lipotoxicity, could play a role in the pathogenesis of liver fibrosis. These results were corroborated by the correlation between circulating SeP with both the soluble macrophage activation marker sCD163 and the non-invasive biomarker of liver fibrogenesis PRO-C3 (r_S_ = 0.33, *p* = 0.021 and r_S_ = 0.30, *p* = 0.033, respectively).

## 3. Discussion

The onset and progression of NAFLD is a complex process involving different metabolic pathways. In this study, we demonstrated for the first time that plasma SeP levels, a selenium carrier protein with antioxidant properties, are related to a deranged metabolic profile and to a severe liver disease in NAFLD subjects without diabetes.

Firstly, our results confirm the relation between circulating SeP and IR, mainly in the adipose tissue. The link between oxidative stress and IR has been described in several studies reporting that an inflamed adipose tissue is able to release several pro-inflammatory factors, such as interleukin-6 (IL-6) and monocyte chemotactic protein-1 (MCP-1), which in turn exacerbate inflammation, IR and oxidative stress, predisposing the development of NASH and diabetes [21]. Despite AT-IR being considered the major determinant in the onset of hepatic fibrogenesis [7,8,9,10], in multivariable regression analysis, SeP remained significantly associated with the degree of liver fibrosis, suggesting that the increased oxidative stress exacerbates liver damage through the worsening of IR. Although in vitro studies performed on HepG2 cells have shown that the transforming growth factor-beta (TGF-β) exerts an inhibitory effect on SeP secretion downregulating *SeP* promoter [22], in vivo evidences that describe a direct link between SeP and hepatic fibrosis in the setting of NAFLD are scarce. If, on one hand, low grade chronic inflammation contributes to the release of pro-inflammatory and pro-fibrogenic cytokines, on the other, IR exacerbates hyperglycemia, which upregulates hepatic expression of SeP in the liver increasing circulating SeP thus establishing a vicious cycle. Conversely, indirect mechanisms encompassing glucose metabolism derangement as well as IR have been proposed. Recently, Chen and colleagues found that circulating SeP significantly increased according to the severity of the disease in a cohort of 79 NAFLD patients [23]. Moreover, the authors showed in vitro that SeP can promote hepatocyte triglyceride overload through the adenosine monophosphate activated protein kinase/acetyl-CoA carboxylase (AMPK/ACC) pathway, one of the key pathogenetic mechanisms leading to the increase in de novo lipogenesis and fatty acids oxidation [23]. Due to the complexity of NAFLD, it is possible that different molecular mechanisms are involved in the regulation of metabolic pathways contributing to the worsening of liver disease; in this context, SeP can be considered an indirect biomarker of the disease status.

Several studies have reported the association between SeP levels and visceral obesity as well as with BMI in NAFLD and overweight patients [16,17]. Although our study cohort included subjects that were not frankly obese, we confirmed the relation between circulating SeP and BMI, suggesting that body weight may influence selenium request by the antioxidant system to counteract the negative effects of oxidative stress.

SeP is the major selenium carrier glycoprotein in plasma and the liver is its main source, although a lesser amount of SeP mRNA was found in other tissues [24,25]. Hepatic expression of SeP is upregulated under hyperglycemic conditions through the transcription complex involving peroxisome proliferator-activated receptor-gamma coactivator 1-alpha (PGC-1α), forkhead box protein O1 alpha (FOXO-1α) and hepatocyte nuclear factor 4 alpha (HNF-4α) [26,27]. Increased SeP levels promote insulin secretion from beta pancreatic cells contributing to hyperinsulinemia and IR; furthermore, SeP knockdown in the liver improves insulin sensitivity [27]. Even if in our cohort we did not find a relation between SeP and glucose levels, we observed an inverse relation between circulating SeP and the glucose rate of disappearance, suggesting that the altered glucose metabolism, in terms of peripheral IR, can modulate stress response in turn promoting SeP expression and release by the liver.

As previously described, AA concentration in blood has been implicated in the pathogenesis of NAFLD [14], and thus we further assessed the relation between circulating SeP and plasma levels of AA to explore the possible link between oxidative stress and the AA profile. We observed a negative correlation between glycine and serine levels with circulating SeP; these data could be explained by the fact that both glycine and serine are involved in the synthesis of glutathione, a molecule with antioxidant properties synthetized in different tissues in response to the increased production of reactive oxygen species (ROS). Increased ROS production, together with inflammation, is a common feature in obese and diabetic patients; in addition, in NAFLD subjects, the increased flux of FFAs from the adipose tissue to the liver, due to the impaired lipolysis caused by AT-IR, determines a fat overload in the hepatocytes, promoting oxidative stress and enhancing SeP synthesis. As previously reported, we confirmed the inverse relation between BCAA levels (leucine, isoleucine, valine) and circulating SeP. Several studies have shown that these AA are implicated in the development of obesity, IR, and diabetes [13,14].

Concerning the lipid profile, we found a positive correlation between circulating SeP and SFA/MUFA levels, specifically with palmitic, margaric and palmitoleic acids. It is well known that the composition of FFAs has an impact on metabolic health. SFAs have a high degree of lipotoxicity and their levels in patients with NAFLD are associated with hepatic steatosis, inflammation, and fibrosis [28]. Moreover, SFAs can activate Kupffer cells in the liver promoting inflammation and, indirectly, fibrogenesis [10]. Our results support the increased request of antioxidant systems by increased levels of SeP to counteract lipotoxic FFAs; these data are also supported by the increased levels of SFAs in patients with severe liver fibrosis even if, in multiple regression analysis, only SeP levels remained significantly associated with the degree of fibrosis (data not shown).

The strength of our paper is represented by the study population, which includes a well characterized group of subjects without diabetes and other potentially confounding metabolic factors. All these patients underwent a liver biopsy and a double-tracer oral glucose tolerance test (OGTT) for the direct measurement of IR components. Furthermore, the analysis of the glucose, lipid and AA profiles confer an added value and allowed us to speculate on the pathogenesis of NAFLD through the hepatokine SeP, which establishes a crosstalk between the liver, the muscle and the adipose tissue. One possible limitation of this study is that the conclusions rely on cross-sectional associations and some of these are weak due to the small number of cases included in the study. Notwithstanding this, our results are consistent with previous findings and are in line with the current literature. Another limitation is the absence of data about the direct measurement of oxidative stress to corroborate the link between SeP levels and ROS production in the setting of IR and NAFLD.

## 4. Materials and Methods

### 4.1. Study Subjects

The study included fifty-seven non-diabetic subjects with NAFLD, selected from consecutive patients who had a liver biopsy between June 2010 and June 2018 in the liver unit of the University of Torino according to the absence of Type 2 diabetes (T2DM). All the patients were tested for the presence of hepatitis B surface antigen, anti-hepatitis B core antigen antibodies and anti-hepatitis C virus antibodies. Other etiologies of liver disease, including viral, autoimmune, cholestatic, genetic, metabolic, alcoholic, and drug-induced were excluded. Past and current ethanol intake <20 g/day had been confirmed through direct questioning of patients and a close relative. A complete medical history and physical examination were undertaken, and anthropometric data were collected at the time of liver biopsy. BMI was calculated on the basis of weight (in kilograms) and height (in meters), waist circumference (to the nearest half-centimeter) was measured at the midpoint between the lower border of the rib cage and the iliac crest.

### 4.2. Histological Features

Liver biopsies were examined by a local expert liver pathologist blinded to patients’ clinical information. The average length of liver tissue was 25 mm (range 14–45 mm), with a minimum of 11 portal tracts. Histological features of NAFLD, such as steatosis, inflammation, ballooning degeneration, and fibrosis, were assessed and scored as described by Kleiner [29]. Diagnosis of NASH was established according to the joint presence of steatosis, ballooning degeneration and lobular inflammation with or without fibrosis.

### 4.3. Analytical Determinations

Serum samples for laboratory investigations were collected at the time of liver biopsy and stored at −80 °C until analysis. Biochemical examinations included the complete blood count, alanine aminotransferase (ALT), aspartate aminotransferase (AST), alkaline phosphatase (ALP) and gamma-glutamyl-transpeptidase (GGT). Serum glucose was measured by the glucose oxidase method (Sentinel, Milan, Italy). Concentrations of free fatty acids (FFAs) were determined by enzymatic colorimetric assays (WAKO diagnostic, Richmond, VA, USA). Total triglycerides, total cholesterol, and high-density lipoprotein cholesterol (HDL) levels were determined by enzymatic colorimetric assays (Sentinel, Milan, Italy). The AA plasma profile was measured by gas chromatography–mass spectrometry (Agilent Technology GC7890-MS5975, Santa Clara, CA, USA) as previously described [14].

### 4.4. Study Design

The study was carried out according to the principles of the Declaration of Helsinki, and it was approved by the ethics committee of the University Hospital “Città della Salute e della Scienza” of Torino (CEI/522, 17 November 2015). All patients gave signed consent for the collection of personal data in the database and for the use of blood samples for research purposes and for participation in the tracer study.

### 4.5. “ In Vivo” Tracer Protocol

All patients underwent a double tracer oral glucose tolerance test (OGTT) within one month of liver biopsy. In vivo Hep-IR and AT-IR were assessed by the stable isotope technique in the fasting state. A primed-continuous infusion of 6,6-D2-glucose (bolus 22 µmol/kg, infusion rate 0.22 µmol/kg min) and of U-2H-glycerol (bolus 1.5 μmol/kg, infusion rate 0.1 μmol/kg min) was administered for 2 h in fasting conditions (equilibration period) to assess endogenous glucose production (EGP) and lipolysis through the rate of appearance of glycerol, as previously described [30,31]. Tracer enrichment of 6,6-D2-glucose and U-2H-glycerol was determined by gas chromatography–mass spectrometry system (5975; Agilent, Palo Alto, CA, USA), selectively monitoring ions at mass to charge ratios of 200, 201, 202, and 205 for glucose and 145, 148 for glycerol. Data were expressed as tracer/trace ratios (TTR) as described [30,31]. Glucose fluxes and glycerol Ra were calculated as infusion rate/TTR. AT-IR was calculated as FFAs*fasting insulin (AT-IR) or as glycerol Ra*fasting insulin (Lipo-IR). Hep-IR was derived from EGP*fasting insulin [31].

### 4.6. Circulating Selenoprotein P Measurement

Blood samples were collected at the time of liver biopsy and stored at −80 °C for further analysis. Plasma levels of SeP were assessed by the commercially available human enzyme linked immunosorbent assay kit (Cloud-Clone Corp. Houston, TX, USA), according to the manufacturer’s instructions. The concentration of SeP was determined with an ELISA reader at 450 nm. The intra- and inter-assay coefficients of variation were below 10% and 12%, respectively. The concentration of SeP was expressed as micrograms per milliliter (μg/mL). Since no cut-off value of SeP has been described to discriminate higher vs. lower circulating levels in non-diabetic subjects with NAFLD, we grouped the whole cohort according to tertile and we used the 75th percentile as cut-off; all the analyses were performed according to this classification.

### 4.7. Statistical Analysis

Data are reported as mean ± standard deviation (SD) for continuous normally distributed variables, as median and range (min–max) for continuous non-normally distributed variables and number and frequency (%) for categorical variables. Comparison between groups was performed with ANOVA or Kruskal–Wallis test as appropriate. For categorical data, the χ^2^ test was used. Spearman correlation was used to assess the correlation between SeP levels with anthropometrical, biochemical, and histological parameters. To determine the association between SeP and the degree of hepatic fibrosis, a multivariable regression analysis was performed. Values of *p* < 0.05 were considered statistically significant. All calculations were performed with MedCalc Software bvba version 18 (Mariakerke, Belgium).

## 5. Conclusions

In conclusion, in this in vivo study performed in a well-characterized cohort of non-diabetic subjects with NAFLD, we observed an interplay between circulating SeP and metabolic derangements, providing a new piece in the complex puzzle of NASH progression and liver fibrogenesis. Our results support the hypothesis that SeP could be a potential target for the amelioration of IR and oxidative stress in patients with NAFLD.

## Figures and Tables

**Table 1:** Anthropometric, biochemical, and histological characteristics of the study cohort according to the cut-off value of selenoprotein P.

Variables	Total *n* = 57	<11.7 ng/mL *n* = 40	≥11.8 ng/mL *n* = 17	*p* Value
Age (years), median (95% CI)	43 (38–48)	42 (36–50)	44 (35–51)	0.794
Male/Female gender, n (%)	44/13 (77/23)	36/4 (90/10)	8/9 (47/53)	<0.001
BMI (kg/m^2^), median (95% CI)	26.8 (25.8–28.8)	25.7 (24.8–27.8)	30.1 (26.8–33)	0.002
Waist circumference (cm), median (95% CI)	95 (91–99)	94 (90–99)	95 (89–103)	0.407
AST (IU/l), median (95% CI)	33 (30–39)	31 (26–36)	56 (31–65)	0.003
ALT (IU/l), median (95% CI)	62 (46–70)	53 (41–70)	63 (47–96)	0.114
Platelets (x 10^9^/l), median (95% CI)	225 (216–246)	227 (215–264)	224 (189–246)	0.589
Fasting glucose (mg/dl), median (95% CI)	93 (90–98)	93 (90–99)	93 (87–108)	0.868
Total cholesterol (mg/dl), median (95% CI)	190 (182–199)	189 (181–200)	194 (169–238)	0.565
HDL cholesterol (mg/dl), median (95% CI)	46 (41–49)	46 (41–49)	48 (31–54)	0.513
Triglycerides (mg/dl), median (95% CI)	100 (89–119)	99 (86–120)	107 (86–215)	0.448
Histological features				
Hepatic steatosis (%), median (range)	32 (21–43)	25 (15–40)	50 (30–65)	0.010
Steatosis score (1/2/3), n (%)	29/20/8 (51/35/14)	24/12/4 (60/30/10)	5/8/4 (29/47/24)	0.033
Ballooning (0/1/2), n (%)	6/27/24 (11/47/42)	5/21/14 (12/53/35)	1/6/10 (6/35/59)	0.107
Lobular inflammation (0/1/2), n (%)	11/43/3 (19/76/5)	7/31/2 (17/78/5)	4/12/1 (23/71/6)	0.708
Fibrosis (0/1/2/3/4), n (%)	20/5/19/11/2 (36/7/30/25/2)	17/5/12/6/0 (42/13/30/15/0)	3/0/7/5/2 (18/0/41/29/12)	0.005
NASH, n (%)	42 (74)	30 (75)	12 (71)	0.732

The cut-off value for selenoprotein P corresponds to the 75th percentile (11.8 ng/mL). Abbreviations: alanine aminotransferase (ALT), aspartate aminotransferase (AST), body mass index (BMI), high-density lipoprotein (HDL), non-alcoholic steatohepatitis (NASH).

**Table 2:** Correlation between selenoprotein P levels and biochemical and metabolic parameters as well as insulin resistance components.

Variables	r_S_	95% CI	*p* Value
Fasting insulin (MU/l)	0.28	0.02–0.50	0.035
Fasting glucose (mg/dl)	−0.05	−0.31–0.21	0.691
Fasting FFAs (mmol/l)	0.42	0.17–0.61	0.001
Glucose clearance (μmol/min × kg)	−0.33	−0.56–−0.06	0.019
Endogenous glucose production (μmol/min × kg)	−0.22	−0.46–0.05	0.106
Glycerol Ra (μmol/min)	−0.34	−0.60–−0.02	0.039
Insulin resistance components			
HOMA-IR	0.26	−0.01–0.49	0.051
Hep-IR (μmol/min × kg × MU/l)	0.27	0.01–0.49	0.043
AT-IR (mmol/l × MU/l)	0.50	0.27–0.67	<0.001
Lipo-IR (μmol/min × MU/l)	0.40	0.15–0.60	0.002

Abbreviations: adipose tissue insulin resistance (AT-IR), alanine aminotransferase (ALT), aspartate aminotransferase (AST), confidence interval (CI), free fatty acids (FFAs), homeostasis model of assessment (HOMA), insulin resistance (IR), rate of appearance (Ra), Spearman coefficient (r_S_).

**Table 3:** Correlation between selenoprotein P levels and the composition of free fatty acids: saturated fatty acids (red), monounsaturated fatty acids (yellow) and polyunsaturated fatty acids (green).

Free Fatty Acids Profile	r_S_	*p* Value	
FFAs (mmol/l)	0.42	0.01	
Palmitic acid (μM)	0.40	0.010	
Stearic acid (μM)	0.33	0.034	SFA
Margaric acid (μM)	0.44	0.005	
Miristic acid (μM)	0.28	0.086	
Palmitoleic acid (μM)	0.45	0.003	
Oleic acid (μM)	0.39	0.011	MUFA
Linoleic acid (μM)	0.25	0.104	
Arachidonic acid (μM)	0.17	0.293	PUFA
SFA (μM)	0.41	0.007	
MUFA (μM)	0.40	0.009	
PUFA (μM)	0.27	0.086	

Abbreviations: free fatty acids (FFAs), monounsaturated fatty acids (MUFA), polyunsaturated fatty acids (PUFA), saturated fatty acids (SFA).

**Table 4:** Univariate and multivariable regression for the identification of the metabolic variables associated with the degree of hepatic fibrosis.

	Univariate Regression Analysis			Multivariable Regression Analysis		
Variables	t	r_p_	*p* Value	t	r_p_	*p* Value
Age (years)	0.928	0.124	0.358	1.217	0.168	0.229
Gender	1.752	0.230	0.085	0.616	0.086	0.541
BMI (kg/m^2^)	3.707	0.447	<0.001	2.763	0.361	0.008
SeP (ng/mL)	3.787	0.455	<0.001	2.369	0.315	0.022
AT-IR (mmol/l * MU/l)	3.215	0.398	0.002	0.599	0.084	0.552

In the regression model, liver fibrosis was considered as an ordinal variable (from 0 to 4). Abbreviations: adipose tissue (AT), body mass index (BMI), insulin resistance (IR), partial correlation coefficient (r_p_), selenoprotein P (SeP).
